# What does it mean when the pleasant smells come and go? Correlation between UPSIT odor identification status and fluctuation of non-motor symptoms in Parkinson’s disease

**DOI:** 10.1007/s13760-025-02727-w

**Published:** 2025-01-22

**Authors:** Hsin-Bei Lei, Ting-Chun Fang, Yu-Hsuan Lin, Shih-Chi Chiu, Ming-Hong Chang, Yi-Jen Guo

**Affiliations:** 1 The Department of Neurological Institute, Taichung Veterans General Hospital, Taichung, Taiwan; 2 Department of Post-Baccalaureate Medicine, College of Medicine, National Chung Hsing University, Taichung, Taiwan; 3 Brain and Neuroscience Research Center, College of Medicine, National Chung Hsing University, Taichung, Taiwan

**Keywords:** Parkinson’s disease, Olfactory fluctuation, UPSIT, Pleasant odors, Non-motor symptoms

## Abstract

Parkinson’s disease (PD) is characterized by motor and non-motor symptoms, including olfactory dysfunction. Prior studies have shown that olfaction deteriorates with disease progression, however fluctuations in olfaction and related PD symptoms have been less explored. This study aimed to investigate correlations between changes in odor identification ability and PD symptoms. PD patients recruited from Taichung Veterans General Hospital underwent at least two consecutive Movement Disorder Society Unified Parkinson’s Disease Rating Scale (MDS-UPDRS) and University of Pennsylvania Smell Identification Test (UPSIT) evaluations. The patients were grouped based on changes in olfactory identification ability between evaluations, and fluctuations in PD symptoms were compared between groups. Ninety-seven PD patients with 114 complete sets of data were analyzed. Significant divergent results were observed between changes in five MDS-UPDRS non-motor subscores and the conversion status of five pleasant odors, including anxiety vs. bubble gum, apathy vs. banana, dizziness vs. coconut, urination vs. root beer, and dopamine dysregulation syndrome (DDS) vs. grape. Fluctuations in the ability to detect pleasant odors, may have a complex interaction with other non-motor symptoms, including in the neurobehavioral and autonomic domains. Serial monitoring of olfactory function, particularly with pleasant odors, may provide valuable insights for tracking non-motor symptoms in PD and warrants further investigation into their therapeutic implications.

## Introduction

Parkinson’s disease (PD) is a neurodegenerative disorder characterized by motor symptoms of bradykinesia, rigidity, resting tremor and postural instability. In the early stages, Lewy bodies pathology involves the olfactory bulb [1], and olfactory dysfunction is a supportive symptom that may precede the onset of initial motor symptoms [2]. Olfactory function has been reported to deteriorate as Parkinson’s disease progresses [3, 4], with particular decline associated with increasing motor scores on the MDS-UPDRS, especially in axial symptoms [5, 6]. Malasa et al. suggested that motor deficits are associated with impairments in both odor identification and odor discrimination, as evaluated by the Sniffin’s Sticks test [7]. Normosmic patients with PD showed significantly fewer motor deficits despite comparable dopamine transporter activity loss in the posterior putamen [8]. Structures related to olfaction, such as the anterior olfactory nucleus and cortical nucleus of the amygdala, as well as neurotransmitters like dopamine and acetylcholine, have been explored in understanding the mechanism of olfactory dysfunction [2]. This implies that olfactory dysfunction could be a biomarker of motor and non-motor progression, encompassing depression, anxiety, autonomic dysfunction, sleep disturbances [7, 9] and cognitive impairment [10, 11].

While olfactory status appears to decline more rapidly in early disease stages [12], some studies have reported a degree of olfactory improvement during follow-ups [13]. Previous studies have reported relationships between non-motor symptoms and specific odors [14–17]. Patients with post-traumatic stress disorder exhibited more aggression, hostility and impulsivity when they showed impaired identification of pleasant odors [14]. Grapefruit and lavender fragrance were described to impact sympathetic or parasympathetic tone [16], while coconut fragrance reduced both sympathetic and parasympathetic response [15]. Few studies have investigated correlations between fluctuations in olfaction with PD symptoms [12, 13, 18]. By investigating the relationships between fluctuations in the ability to detect each odor in the University of Pennsylvania Smell Identification Test (UPSIT) and PD motor and non-motor symptoms, this study aims to increase the awareness of clinicians regarding the evaluation and timely management of possible PD-related symptoms in patients with fluctuations in the ability to identify specific odors.

## Materials and methods

### Subjects

This retrospective study was conducted between October 2016 and April 2022, and recruited PD patients from the Center for Parkinson and Movement Disorders at Taichung Veterans General Hospital (VGHTC). All patients met the clinically probable PD diagnostic criteria outlined in the International Parkinson and Movement Disorder Society Clinical Diagnostic Criteria for Parkinson’s Disease. Clinical evaluations, including the Chinese version of the UPSIT [19], Movement Disorder Society Unified Parkinson’s Disease Rating Scale (MDS-UPDRS) [20], Hoehn and Yahr (HY) Scale [21], Montreal Cognitive Assessment (MoCA) [22, 23], and Beck Depression Inventory–II (BDI-II) [24], were performed when the patients were on medication, and the data collected for analysis is recorded during ‘on’ status. The levodopa equivalent daily dosage (LEDD) was calculated for all participants [25]. The final analysis included only those who completed at least two UPSIT and MDS-UPDRS evaluations with an interval of 12–24 months. The study received approval from the Institutional Review Board of Taichung Veterans General Hospital (Approval No.: CE22189B-1), and all personal information was encrypted to ensure patient privacy.

### Olfactory testing

The Chinese version of the UPSIT was used to assess olfactory identification function [19]. The test comprises 40 items, including pizza, bubble gum, methanol, cherry, motor oil, mint, banana, sandalwood, leather, coconut, onion, fruit juice, licorice, fish, coffee, gasoline, strawberry, cedar, chocolate, rubber tire, lilac, turpentine, peach, root beer, jasmine, pineapple, grapefruit, orange, magnolia, watermelon, paint thinner, baby powder, smoke, pine, grape, lemon, soap, natural gas, rose, and peanut. On each page, a sniff strip was scratched to release the embedded odorant, and the participants were then asked to identify the correct odor from four choices. In previous research [26], the cut-off values of Chinese version of the UPSIT were established at ≤ 29.5 as detecting hyposmia in early PD patients with less than 2 years of disease duration.

### Study design

#### Patient grouping according to the ability to identify the same odor between two consecutive UPSIT examinations

**Fig. 1:**
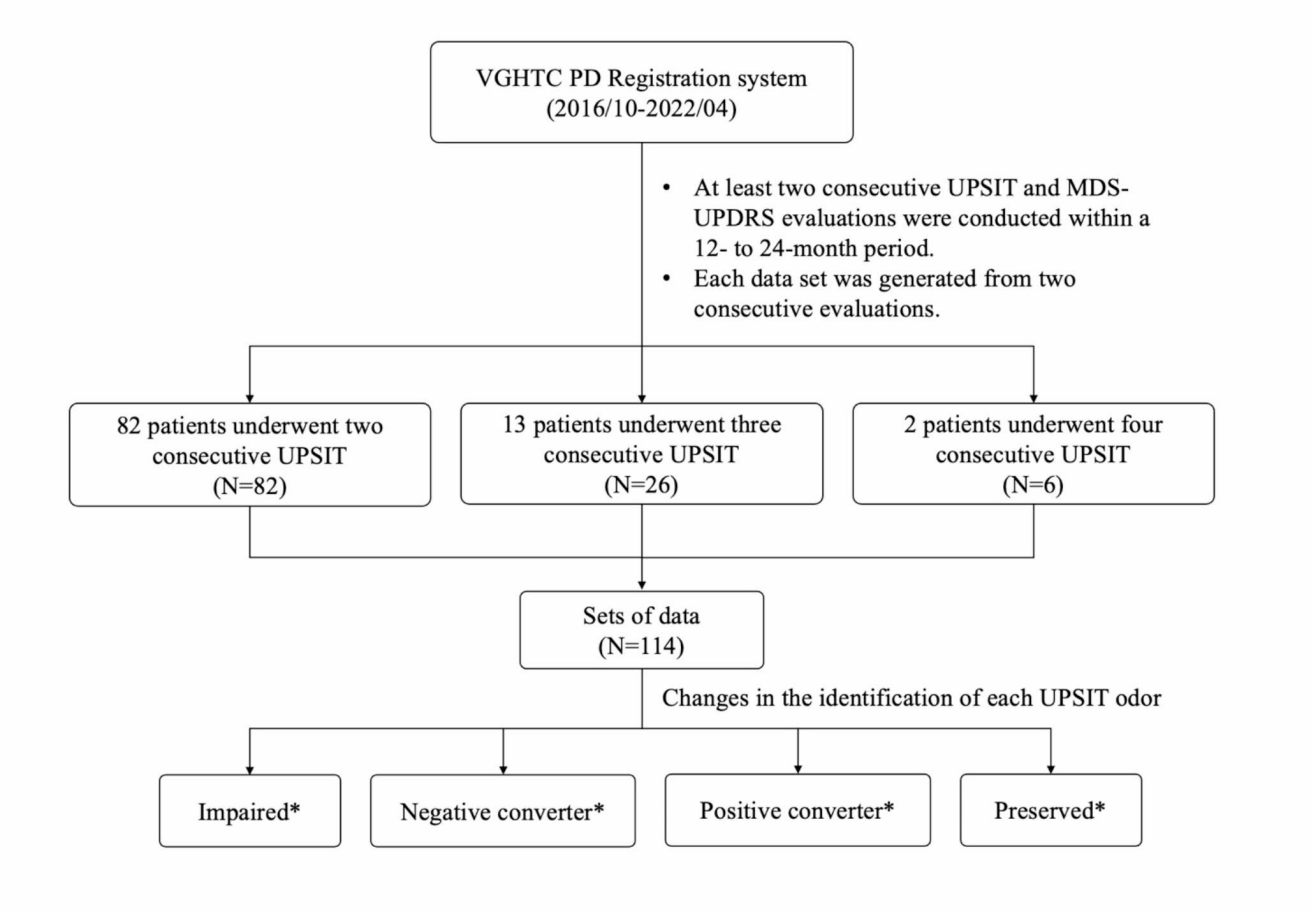
This analysis included 97 PD patients who completed at least two UPSIT and MDS-UPDRS evaluations within a 12- to 24-month period. Of these, 82 patients completed two evaluations, providing equivalent data sets; 13 patients completed three evaluations, generating 26 data sets; and 2 patients completed four evaluations, resulting in 6 data sets. In total, 114 data sets (*N* = 114) from 97 patients were analyzed. Based on odor identification results from the consecutive UPSIT tests, patients were categorized into four groups: impaired, preserved, positive converters, and negative converters. Patients who incorrectly identified the same odor in both tests were classified as impaired, while those who identified it correctly in both tests were categorized as preserved. Positive converters were those who initially misidentified an odor but later identified it correctly, while negative converters were those who initially identified it correctly but later misidentified it. A patient could be classified into different groups for different odors * The number of data points in each group varies according to the sequential differences in odor identification for each specific odor

According to the identification results of each odor in the two consecutive UPSIT tests, the patients were classified into four groups: impaired, positive converter, negative converter, and preserved. Patients who wrongly identified the same odor in the two consecutive UPSIT tests were classified into the “impaired” group, while those who correctly identified the same odor in both UPSIT tests were classified into the “preserved” group. The “positive converter” group referred to patients who failed to identify a particular odor in one UPSIT test, but who correctly identified the same odor in the subsequent test. The “negative converter” group consisted of patients who correctly identified a particular odor in one UPSIT test, but who failed to identify the same odor in the subsequent UPSIT test. The same patient could therefore be classified into different groups for different UPSIT odors. The flowchart for study design is shown in Fig. 1.

#### Exploring the association between changes of MDS-UPDRS non-motor subscores and UPSIT odor identification conversion status

Changes in each item of MDS-UPDRS part 1 score and MDS-UPDRS part 3 score between the two MDS-UPDRS examinations were calculated, and differences were compared among the four groups. For the odors with significant differences among the four groups, post-hoc comparisons were done between groups. To resolve problems of data heterogeneity, the baseline demographic variables of each comparison was checked in the four study groups for the odors showing significant differences between the negative and positive converter groups, including age, gender, disease duration, follow-up interval, education years, MoCA, BDI-II, MDS-UPDRS part 1 to part 4 scores, and LEDD. Generalized Estimating Equations (GEEs) were employed to investigate the associations between changes in MDS-UPDRS subscores and UPSIT odor identification conversion status, taking into account various sets of repeated measures among participants. If the changes in MDS-UPDRS non-motor subscores showed a divergent result in the positive and negative converter groups, with significant p values in the GEEs, the MDS-UPDRS non-motor subitem was considered to have an impact on the conversion status of the UPSIT odor.

### Statistical analysis

All data were analyzed using SPSS version 23.0 (IBM Inc., Armonk, NY). The Kruskal-Wallis test was used for continuous variables due to small sample size in each group, and the chi-square test was used for categorical variables. Pairwise comparisons following the Kruskal-Wallis test were conducted using Dunn’s post hoc tests in SPSS. Generalized estimating equations (GEEs) were utilized to explore the relationships between changes in MDS-UPDRS subscores and odor identification status, categorized as negative or positive converters. The chosen parameters included a model-based estimator, unstructured correlation matrix, linear model, and a model with the main effect only. Changes in MDS-UPDRS Part 1 subscores served as dependent variables, with positive versus negative conversion status of UPSIT subitems as factors. The order of repeated measurements was included as covariates for statistical analysis. A p-value of < 0.05 was considered statistically significant.

## Results

### Demographic data of all participants at study entry

The demographic data of all participants at study entry are shown in Table 1. In this study, ninety-seven PD patients were recruited. Changes of odor identification status from every two evaluations were recorded to be one set of data. Eighty-two patients underwent two evaluations and generated an equivalent amount of data. Thirteen patients underwent three assessments, resulting in twenty-six sets of data. Two patients received four evaluations and generated six sets of data for comparison. With this strategy, 114 sets of data (*N* = 114) collected from ninety-seven PD patients were enrolled for statistical analysis. The average age of the participants was 67.06 ± 8.67 years, and 64.9% were male. The olfactory identification tests were performed an average 6.03 ± 4.37 years from the time of the initial diagnosis, and the average score was 16.06 ± 6.67. The mean interval between the two assessments was 16.04 ± 3.58 months. The initial average MoCA, BDI-II, MDS-UPDRS total and part I-IV scores were 24.48 ± 4.81, 11.04 ± 8.65, 51.02 ± 22.97, 9.69 ± 6.14, 9.61 ± 7.37, 30.55 ± 12.73, and 1.16 ± 2.27, respectively, and the initial average LEDD was 546.66 ± 432.42 mg/day.

**Table 1:** Demographic data of PD patients while entry of the study

PD registration databank (*N* = 114)	
Age, years-old (Mean ± SD)	67.06 ± 8.67
Gender, Male (N (%))	74 (64.9)
Disease duration, years (Mean ± SD)	6.03 ± 4.37
Interval between follow-up, months (Mean ± SD)	16.04 ± 3.58
MoCA (Mean ± SD)	25.47 ± 4.03
BDI-II (Mean ± SD)	11.04 ± 8.65
MDS-UPDRS total (Mean ± SD)	51.02 ± 22.97
MDS-UPDRS part 1 (Mean ± SD)	9.69 ± 6.14
MDS-UPDRS part 2 (Mean ± SD)	9.61 ± 7.37
MDS-UPDRS part 3 (Mean ± SD)	30.55 ± 12.73
MDS-UPDRS part 4 (Mean ± SD)	1.16 ± 2.27
LEDD, mg/day (Mean ± SD)	546.66 ± 432.42
UPSIT (Mean ± SD)	16.06 ± 6.67

PD: Parkinson’s disease; MoCA: Montreal Cognitive Assessment; BDI-II: Beck Depression Inventory-II; MDS-UPDRS: Movement Disorder Society-sponsored revision of the Unified Parkinson’s Disease Rating Scale; LEDD: Levodopa equivalent daily dosage; UPSIT: University of Pennsylvania Smell Identification Test

### UPSIT odor conversion status and its relationship to non-motor symptoms

In the two consecutive UPSIT assessments, while 43.77% and 23% sets of consecutive data remained in the impaired and preserved groups, respectively, an average of 17.26% exhibited negative conversion and 15.97% exhibited positive conversion in any of the UPSIT odors.

Table 2 shows the results with significant differences in MDS-UPDRS non-motor subscore changes regarding UPSIT odor identification conversion status between the two consecutive assessments. Changes in MDS-UPDRS 1.4 anxiety score were significantly different among the four UPSIT bubble gum and strawberry odor identification conversion status groups (△MDS-UPDRS 1.4 anxiety scores of −0.04 ± 0.68, −0.4 ± 0.76, 0.4 ± 0.82, and − 0.29 ± 0.85 in the bubble gum impaired, negative converter, positive converter and preserved groups, respectively, *p* = 0.012; △MDS-UPDRS 1.4 anxiety scores of 0.51 ± 0.62, 0.78 ± 0.73, 0.62 ± 0.65, and 0.25 ± 0.5 in the strawberry impaired, negative converter, positive converter and preserved groups, respectively, *p* = 0.011). Changes in MDS-UPDRS 1.5 apathy score showed significant differences regarding UPSIT banana odor identification conversion status (△MDS-UPDRS 1.5 apathy scores of 0.04 ± 0.94, 0.43 ± 0.87, −0.33 ± 0.83, and − 0.29 ± 0.77 in the impaired, negative converter, positive converter and preserved groups, respectively, *p* = 0.031). Changes in MDS-UPDRS 1.10 urination score and MDS-UPDRS 1.12 dizziness score were different according to UPSIT coconut odor identification conversion status (△MDS-UPDRS 1.10 urination score of −0.15 ± 1.09, −0.14 ± 1.01, 0.53 ± 1.28, and − 0.78 ± 0.97 in the impaired, negative converter, positive converter and preserved groups, respectively, *p* = 0.049; △MDS-UPDRS 1.12 dizziness scores of −0.03 ± 1.11, −0.62 ± 1.12, 0.59 ± 1.06, and − 0.22 ± 0.97 in the impaired, negative converter, positive converter and preserved groups, respectively, *p* = 0.008). Changes in MDS-UPDRS 1.6 dopamine dysregulation syndrome (DDS) score were different in the four UPSIT onion and grape odor identification conversion status groups (△MDS-UPDRS 1.6 DDS scores of 0.06 ± 0.70, −0.08 ± 0.41, 0.69 ± 1.40, and − 0.15 ± 0.57 in the onion impaired, negative converter, positive converter and preserved groups, respectively, *p* = 0.013; △MDS-UPDRS 1.6 DDS scores of 0.06 ± 0.87, 0.43 ± 0.76, −0.29 ± 0.69, and 0 ± 0.37 in the grape impaired, negative converter, positive converter and preserved groups, respectively, *p* = 0.021). Changes in MDS-UPDRS 1.2 psychosis, 1.10 urination, and 1.11 constipation severity scores differed according to UPSIT watermelon, root beer, and chocolate odor identification conversion status, respectively (△MDS-UPDRS 1.2 psychosis scores of 0.1 ± 0.58, −0.27 ± 0.46, 0.13 ± 0.45, and − 0.04 ± 0.20 in the watermelon impaired, negative converter, positive converter and preserved groups, respectively, *p* = 0.032; △MDS-UPDRS 1.10 urination scores of 0.26 ± 0.89, −0.62 ± 1.14, 0.29 ± 1.16, and − 0.21 ± 1.16 in the root beer impaired, negative converter, positive converter and preserved groups, respectively, *p* = 0.004; △MDS-UPDRS 1.11 constipation scores of −0.68 ± 0.95, 0.41 ± 0.87, 0.2 ± 0.78, and 0.21 ± 0.92 in the chocolate impaired, negative converter, positive converter and preserved groups, respectively, *p* = 0.002).

**Table 2:** Significant differences of MDS-UPDRS non-motor subscore changes regarding to UPSIT subodor identification conversion status during follow-up

MDS-UPDRS subitem	UPSIT odor identification conversion status				
	Impaired	Negative converter	Positive converter	Preserved	*p* value
1.4 Anxiety^	**UPSIT sub-item: Bubble gum**				
Case number (N, %)	48	25	20	21	*p* = 0.012
△ score (mean ± SD)	−0.04 ± 0.68	−0.4 ± 0.76	0.4 ± 0.82	−0.29 ± 0.85	
1.5 Apathy^	**UPSIT sub-item: Banana**				
Case number (N, %)	49	21	27	17	*p* = 0.031
△ score (mean ± SD)	0.04 ± 0.94	0.43 ± 0.87	−0.33 ± 0.83	−0.29 ± 0.77	
1.12 Dizziness^	**UPSIT sub-item: Coconut**				
Case number (N, %)	67	21	17	9	*p* = 0.008
△ score (mean ± SD)	−0.03 ± 1.11	−0.62 ± 1.12	0.59 ± 1.06	−0.22 ± 0.97	
1.10 Urination^^^	**UPSIT sub-item: Coconut**				
Case number (N, %)	67	21	17	9	*p* = 0.049
△ score (mean ± SD)	−0.15 ± 1.09	−0.14 ± 1.01	0.53 ± 1.28	−0.78 ± 0.97	
1.6 DDS^^^	**UPSIT sub-item: Onion**				
Case number (N, %)	33	24	16	41	*p* = 0.013
△ score (mean ± SD)	0.06 ± 0.70	−0.08 ± 0.41	0.69 ± 1.40	−0.15 ± 0.57	
1.4 Anxiety*	**UPSIT sub-item: Strawberry**				
Case number (N, %)	79	18	13	4	*p* = 0.011
△ score (mean ± SD)	0.51 ± 0.62	0.78 ± 0.73	0.62 ± 0.65	0.25 ± 0.50	
1.11 Constipation*, ***	**UPSIT sub-item: Chocolate**				
Case number (N, %)	19	17	15	63	*p* = 0.002
△ score (mean ± SD)	−0.68 ± 0.95	0.41 ± 0.87	0.2 ± 0.78	0.21 ± 0.92	
1.10 Urination^	**UPSIT sub-item: Root beer**				
Case number (N, %)	31	24	17	42	*p* = 0.004
△ score (mean ± SD)	0.26 ± 0.89	−0.62 ± 1.14	0.29 ± 1.16	−0.21 ± 1.16	
1.2 Psychosis^	**UPSIT sub-item: Watermelon**				
Case number (N, %)	50	15	24	25	*p* = 0.032
△ score (mean ± SD)	0.1 ± 0.58	−0.27 ± 0.46	0.13 ± 0.45	−0.04 ± 0.20	
1.6 DDS^	**UPSIT sub-item: Grape**				
Case number (N, %)	67	14	17	16	*p* = 0.021
△ score (mean ± SD)	0.06 ± 0.87	0.43 ± 0.76	−0.29 ± 0.69	0 ± 0.37	

MDS-UPDRS: Movement Disorder Society-sponsored revision of the Unified Parkinson’s Disease Rating Scale; DDS: Dopamine Dysregulation Syndrome; UPSIT: University of Pennsylvania Smell Identification Test; ∆: change between two evaluations; *Impaired vs. Negative converter, *p* < 0.05, **Impaired vs. Positive converter, *p* < 0.05, ***Impaired vs. Preserved, *p* < 0.05, ^Negative converter vs. Positive converter, *p* < 0.05, ^^Negative converter vs. Preserved, *p* < 0.05, ^^^Positive converter vs. Preserved, *p* < 0.05

### Significant divergence in non-motor symptom severity fluctuations in the negative and positive UPSIT odor converters

**Fig. 2:**
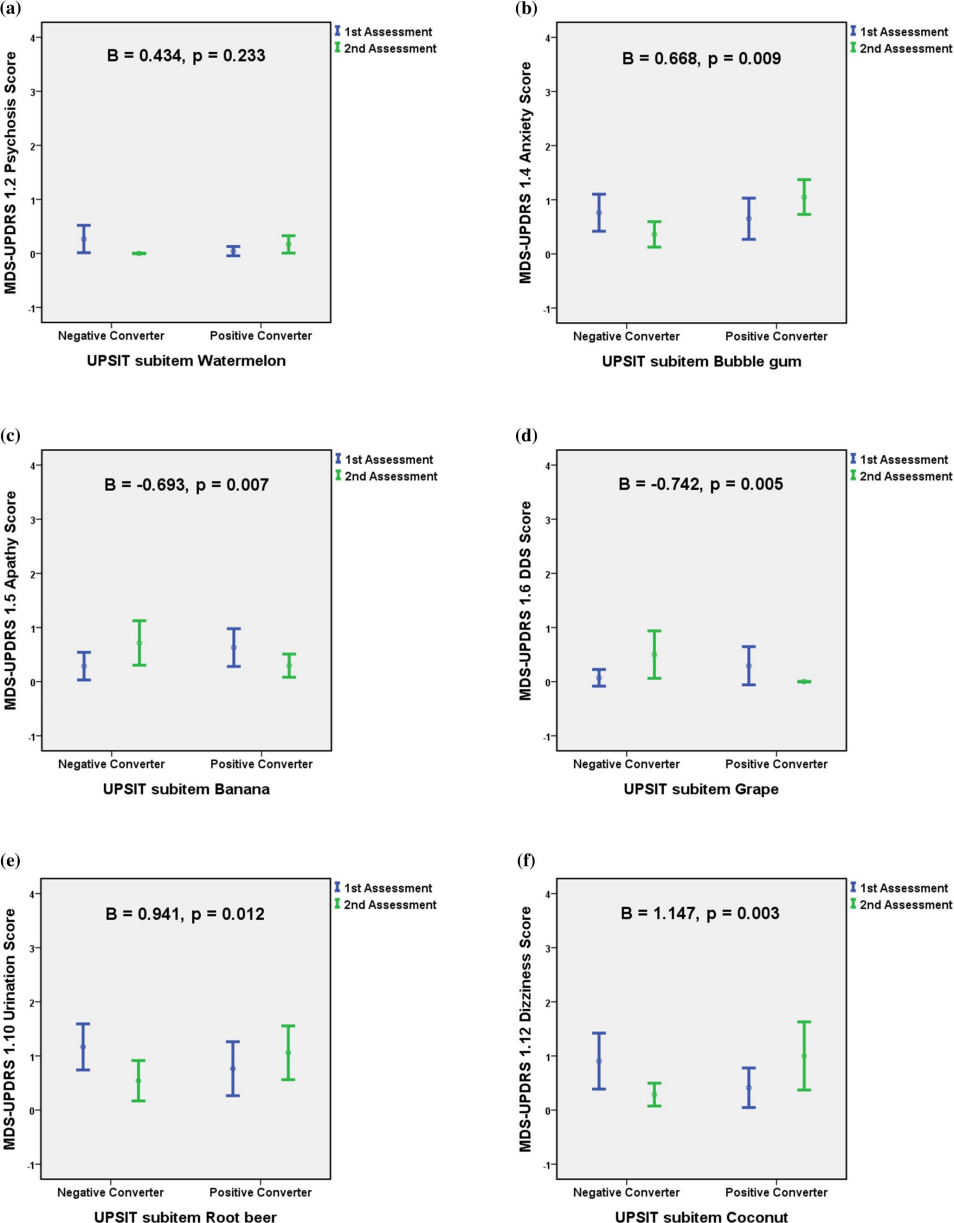
Fluctuations in MDS-UPDRS non-motor symptoms and the relationships between UPSIT odor positive converters and negative converters. The upper and lower borders of each error bar represented the 95% confidence interval of the MDS-UPDRS non-motor symptom subscore. The B and p values were computed using the generalized estimating equations. Serial changes in significant differences of MDS-UPDRS non-motor subscores with corresponding UPSIT odor identification conversion status between the first and second assessment revealed: **(a)** MDS-UPDRS 1.2 psychosis score vs. UPSIT watermelon odor identification: B = 0.434, *p* = 0.233; **(b)** MDS-UPDRS 1.4 anxiety score vs. UPSIT bubble gum odor identification: B = 0.668, *p* = 0.009; **(c)** MDS-UPDRS 1.5 apathy score vs. UPSIT banana odor identification: B=−0.693, *p* = 0.007; **(d)** MDS-UPDRS 1.6 DDS score vs. UPSIT grape odor identification: B=−0.742, *p* = 0.005; **(e)** MDS-UPDRS 1.10 urination score vs. UPSIT root beer odor identification: B = 0.941, *p* = 0.012; **(f)** MDS-UPDRS 1.12 dizziness score vs. UPSIT coconut odor identification: B = 1.147, *p* = 0.003. Blue line: first assessment; Green line: second assessment; UPSIT: University of Pennsylvania Smell Identification Test; MDS-UPDRS: Movement Disorder Society-sponsored revision of the Unified Parkinson’s Disease Rating Scale; DDS: Dopamine Dysregulation Syndrome

Post-hoc comparisons of those showing significant differences in MDS-UPDRS non-motor subscore changes regarding UPSIT odor identification conversion status between the two assessments disclosed significantly divergent results in MDS-UPDRS non-motor symptom changes between specific positive and negative odor converters, including: △MDS-UPDRS 1.2 psychosis score vs. watermelon odor (*p* = 0.032), △MDS-UPDRS 1.4 anxiety score vs. bubble gum odor (*p* = 0.012), △MDS-UPDRS 1.5 apathy score vs. banana odor (*p* = 0.031), △MDS-UPDRS 1.6 DDS score vs. grape odor (*p* = 0.021), △MDS-UPDRS 1.10 urination score vs. root beer (*p* = 0.004), and △MDS-UPDRS 1.12 dizziness score vs. coconut (*p* = 0.008) (Table 2). There were no significant differences in baseline demographic data including age, disease duration, follow-up interval, education years, MoCA, BDI-II, and MDS-UPDRS part 1 to part 4 scores, and LEDD between the positive and negative converters of these six odors. An increase in anxiety, dizziness, urination, and psychosis scores was associated with improved olfactory function for bubble gum, coconut, root beer, and watermelon odors. However, positive converters of banana and grape exhibited decreasing apathy and DDS scores between the two assessments. (Fig. 2; Table 3). The GEE analysis, considering the number of repeated measurements for each participant, revealed statistically significant relationships between MDS-UPDRS non-motor symptoms and specific UPSIT odor identification conversion status, including MDS-UPDRS 1.4 anxiety score vs. bubble gum (Beta = 0.668, *p* = 0.009), MDS-UPDRS 1.5 apathy score vs. banana (Beta=−0.693, *p* = 0.007), MDS-UPDRS 1.6 DDS score vs. grape (Beta=−0.742, *p* = 0.005), MDS-UPDRS 1.10 urination score vs. root beer (Beta = 0.941, *p* = 0.012), and MDS-UPDRS 1.12 dizziness score vs. coconut (Beta = 1.147, *p* = 0.003) (Fig. 2; Table 3).

**Table 3:** Relationship between dynamics of odor identification and fluctuation of PD non-motor symptoms

Detection of UPSIT odor	Emotional valence of odor	MDS-UPDRS Part I item	Non-motor classification	Non-motor symptom subscore	B	*p* value
Watermelon	Pleasant	1.2 (Psychosis)	Neurobehavior	Increase	0.434	0.233
Bubble gum	Pleasant	1.4 (Anxiety)	Neurobehavior	Increase	0.668	**0.009**
Banana	Pleasant	1.5 (Apathy)	Neurobehavior	Decrease	−0.693	**0.007**
Grape	Pleasant	1.6 (DDS)	Neurobehavior	Decrease	−0.742	**0.005**
Root beer	Pleasant	1.10 (Urination)	Autonomic	Increase	0.941	**0.012**
Coconut	Neutral/Pleasant	1.12 (Dizziness)	Autonomic	Increase	1.147	**0.003**

B and p values computed using the generalized estimating equations; PD: Parkinson’s disease; UPSIT: University of Pennsylvania Smell Identification Test; MDS-UPDRS: Movement Disorder Society-sponsored revision of the Unified Parkinson’s Disease Rating Scale; DDS: Dopamine Dysregulation Syndrome

## Discussion

### Fluctuations of odor identification ability, especially pleasant odors, may be related to changes in non-motor symptoms in PD patients

The main findings of this study were the significant correlations between fluctuations in non-motor symptoms and the ability to identify specific pleasant odors. While positive converters of bubble gum, coconut, root beer and watermelon showed increasing severity of anxiety, dizziness, and urination, respectively, negative converters of these odors showed divergent results regarding fluctuations in these non-motor symptoms. In contrast, compared with the negative converters, the positive converters of banana and grape showed decreasing severity of apathy and DDS.

### Negative pleasant odor identification conversion may indicate worsening of apathy and DDS

Previous studies discussing the relationship between hyposmia and apathy have reported conflicting results [7, 27–29]. While one article did not identify a correlation between apathy and olfactory dysfunction [29], another study suggested that reduced olfactory stimulation for a long-period may reduce emotional memory as well as emotional responses to the external environment, resulting in apathy to external stimuli [28]. Another study reported that chronic schizophrenia patients, with more negative symptoms, had worse pleasant odor identification ability than controls [30]. In the present study, we also found that failure to identify the pleasant odor banana was associated with worsening of apathy symptoms, and vice versa, in PD patients. Grape and banana odorants have been associated with improvements in neurobehavioral symptoms. Grape has demonstrated protective effects against oxidative damage in a rat model, as noted in a systematic review [31], and has been shown to restore reactive oxygen species levels in a rotenone-induced in vitro PD model [32]. Another study found that grape juice improved motor symptoms following apomorphine treatment when compared to an exercise group [33]. Bananas, known for their antioxidant properties, are rich in amine compounds such as dopamine and tryptophan [34], which increase parasympathetic activity [35]. Additionally, banana consumption has been linked to reduced depressive symptoms in males [36]. The antioxidative properties of both grapes and bananas may help reduce the clinical severity of non-motor symptoms.

Functional associations between DDS, such as atypical or excessive gambling, sexual interests, repetitive hobbies or medication addiction, and the orbitofrontal cortex have been reported in previous literature [14, 37], and patients with hyposmia have been associated with a higher prevalence of DDS [37–39]. In addition, pleasant odors have been reported to be more likely to activate the orbitofrontal cortex and exhibit a stronger association with impulsivity compared to unpleasant or neutral odors [14]. This supports our finding that fluctuations in the ability to identify the pleasant odor grape were associated with a higher DDS score in the MDS-UPDRS. In summary, poor odor identification ability indicated decreased inhibitory control [38].

### Detecting pleasant odors may not be good for autonomic function

While our findings align with prior hyposmia research discussing apathy and DDS, dysfunction in several autonomic domains, including dizziness and urinary incontinence, were associated with improvements in pleasant coconut and root beer odor perception, respectively, in the current study. These findings appear to conflict with prior research [9, 40, 41]. Iijima et al. reported that the cardiac sympathetic system degenerates in parallel with olfactory function during the early stages of PD, while in advanced stages it may become more complicated [42]. This may at least partially explain the inconsistency between our results and other studies. While previous literature has focused on exploring the relationship between total scores of olfactory tests and PD autonomic symptoms, our study further explored the relationships between the ability to detect each UPSIT odor and PD non-motor symptoms. Different odors may have different effects on the autonomic system. For example, grapefruit odor bas been reported to increase sympathetic tone, while lavender odor has been reported to decrease sympathetic tone [16]. In addition, inhaling coconut fragrance during the Stroop color-word task has been shown to enhance blood pressure recovery, possibly through sympathetic pathways, resulting in lower blood pressure surge when facing stressors [15]. This would be detrimental to the dizziness symptoms in PD patients, since they tend to have orthostatic hypotension. Root beer is known for its diuretic effects, promoting urination [43]. Few studies have discussed micturition and olfactory cues in humans. In a mouse model, the combined effect of olfaction and social hierarchy was shown to affect micturition patterns in adult male mice [44]. Further studies are needed to investigate the pathophysiological relationship between micturition function and olfactory impairment in PD patients.

### Positive odor identification converters potentially had more anxiety and psychosis symptoms

In contrast to the negative converters, the positive converters of bubble gum odor showed increasing MDS-UPDRS anxiety scores in this study. Clinically, bubble gum has been used to alleviate anxiety in stressful situations and has been shown to improve self-focused attention [45, 46]. While several studies have indicated a connection between a higher state of anxiety and decreased olfactory function [17, 47, 48], some studies reported contrary results [49, 50], suggesting that increased anxiety may lead to faster odor identification, and that individuals with prolonged hyposmia or anosmia tend to exhibit lower levels of anxiety.

Regarding psychosis, our study revealed a contrasting result compared to previous studies [51]. There is currently no existing research supporting our findings or illustrating the potential mechanisms behind them. Although none of the PD patients in our study reported experiencing olfactory hallucinations during assessments with the MDS-UPDRS part 1.2 and Neuropsychiatric Inventory–Questionnaire, one study reported that among PD patients with olfactory hallucination, 81.3% experienced pleasant smells such as flowers and fruits [52], which may suggest the possibility of higher sensitivity to pleasant smells in those patients. Since the statistical significance was not retained in the GEE analysis considering repeated measurements of the participants in our study, further investigations to examine the relationships and underlying reasons are warranted.

### Strengths and limitations of this study

While most prior studies have been cross-sectional, this study provides longitudinal observations about the relationship of each UPSIT odor identification ability with fluctuations in non-motor symptoms. There were also several intrinsic limitations, including the small sample size, which limited further grouping for comparison, lack of healthy volunteers, differences in disease severity of the participants on study entry, and that the precise pathophysiological mechanisms of some findings remain unclear. In addition, the result of our UPSIT result may be affected by cultural differences in some degree, despite eight odors were replaced during validation of Chinese version of UPSIT [19]. The composition of odors is an important consideration, as monomolecular odors are determined by concentration and chemical structure, whereas multimolecular odors are influenced by more complex mixtures [53]. However, all 40 odors in the Chinese version of the UPSIT test consist of multiple molecular components, indicating their multimolecular nature. This limits the ability to investigate the impact of monomolecular versus multimolecular odor loss on PD clinical symptoms. Further large-scale investigations utilizing neurophysiological or imaging tools may further confirm our results and provide a clearer picture about the underlying pathogeneses.

## Conclusions

Olfactory dysfunction worsens during the course of PD. Fluctuations in odor identification ability, and especially pleasant odors, may have complex interactions with other non-motor symptoms, including in the neurobehavioral and autonomic domains. Serial follow-up of olfactory function, especially pleasant odors, is suggested when treating PD patients. In addition, in patients with fluctuations in odor identification ability, clinicians should be aware of possible related and treatable non-motor symptoms.

## Data Availability

This data utilized in this study were extracted from the Registration Platform for Cognitive Function in Parkinsonism Patients of Taichung Veterans General Hospital. The datasets generated during and/or analyzed during the current study are available from the corresponding author on reasonable request.
